# Influence of Lakeshore Riparian Vegetation on Diet, Feeding Rate, and Body Condition of Adfluvial Coastal Cutthroat Trout

**DOI:** 10.1002/ece3.71470

**Published:** 2025-06-02

**Authors:** Tracy Michalski, Heather Klassen, Peter Ott, Carl Schwarz

**Affiliations:** ^1^ Coast Area Research British Columbia Ministry of Forests Nanaimo British Columbia Canada; ^2^ Forest Analysis and Inventory Branch British Columbia Ministry of Forests Victoria British Columbia Canada; ^3^ StatMathComp Consulting Port Moody British Columbia Canada

**Keywords:** aquatic‐terrestrial coupling, forest harvest, littoral zone, *Oncorhynchus clarkii clarkii*, trophic ecology

## Abstract

Terrestrial invertebrates provide energy and nutrients to lacustrine systems. However, the extent to which lakeshore and riparian vegetation affects the diet of lake‐associated fish is not well known. We sampled six small lakes (< 1 km^2^ surface area) on the West Coast of British Columbia, Canada, to determine if lakeshore riparian vegetation composition and extent affected the diet, feeding rate, and body condition of adfluvial cutthroat trout ( *Oncorhynchus clarkii clarkii* ). We found strong evidence that cutthroat sampled from a lake with an intact, old forest riparian had a different diet composition comprised largely of terrestrial invertebrates than cutthroat sampled from lakes with riparian forests representing a gradient of vegetation age and cover. We identified positive relationships between the intake of terrestrial invertebrates by cutthroat with the percentage of riparian vegetation overhanging and submerged along and decaying wood within the littoral zone. We also found positive relationships between the percentage of vegetation overhanging and submerged along the littoral zone and the percentage of overstory terrestrial vegetation. Our study contributes to a growing body of evidence recognizing the connections between upland terrestrial and lakeshore riparian and aquatic ecosystems.

## Graphical abstract

Our research compares the diet, feeding rates, and condition of Coastal cutthroat trout in six small (1 km^2^ area) lakes with lakeshore riparian zones representing a gradient of logging histories. We identified positive relationships between the intake of terrestrial invertebrates by cutthroat and the percentage of riparian vegetation overhanging and submerged along, and decaying wood within the littoral zone. We also found positive relationships between the percentage of vegetation overhanging and submerged along the littoral zone and the percentage of overstory terrestrial vegetation.

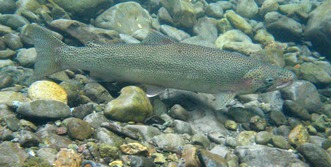

## 1 Introduction

Lakes are complex ecosystems and are linked to their riparian landscapes by the flow of energy and nutrients produced outside the boundaries of the aquatic habitat. Accumulating evidence from North America (Craig et al. 2015; Emery 2015; Tanentzap et al. 2014; Cole et al. 2011; Solomon et al. 2011), Europe (Mehner et al. 2016; Karlsson and Byström 2005; Grey et al. 2001; Bartels et al. 2012), and Asia (Kawaguchi et al. 2003; Yoshii et al. 1999) now suggests that the use of terrestrially derived resources can be as high as 40%–94% in some lake food webs (Tanentzap et al. 2017). The lakeshore riparian zone is the transition between the shallow, nearshore lake littoral zone, and the surrounding terrestrial environment. As the interface between the upland terrestrial and lake ecosystems, the lakeshore riparian ecosystem plays a critical role in nutrient cycling and productivity. Additionally, the lakeshore littoral zone facilitates the transfer of resources, energy, and nutrients between the terrestrial and aquatic ecosystems (Naiman et al. 1998; Wetzel 2001; Gratton et al. 2008; Pusey and Arthington 2003). Given the number of important relationships between riparian and aquatic ecosystems, it is not surprising that spatial and temporal variation in adfluvial fish assemblage, composition, and characteristics have been linked to variation in riparian cover (Pusey and Arthington 2003). There is increasing evidence that fish communities are adversely affected by riparian alteration and recover only when riparian integrity is reestablished (Penczak 1995).

Terrestrial invertebrates serve as bioenergetic links between terrestrial and aquatic food webs, supply high‐quality diet items that form a substantial part, at least seasonally, of the diet of salmonids and can strongly influence the diversity and biomass of fish assemblages (Kawaguchi et al. 2003; Hunt and Krokhin 1975; Cada et al. 1987; Nielsen 1992; Syrjänen et al. 2011; Studinski and Hartman 2015; Ramey and Richardson 2017; Niles and Hartman 2021; Torres Bejarano 2021). For example, Saunders and Fausch (2012) found that terrestrial invertebrates contributed about 30%–45% of the biomass of prey in trout diets when averaged across stream systems in Colorado. Nielsen (1992) found that terrestrially derived prey composed up to 28% of the total energy intake of juvenile coho salmon ( *Oncorhynchus kisutch* [Walbaum, 1792]) in Washington, and Wipfli (1997) reported that terrestrial prey composed over half of the biomass ingested by Dolly Varden char ( *Salvelinus malma* [Walbaum, 1792]), juvenile coho salmon, and cutthroat trout ( *Oncorhynchus clarkii clarkii* [Richardson, 1836]) in several southeastern Alaska streams. Studies limiting terrestrial prey in streams have shown declines in the growth of Dolly Varden char (Baxter et al. 2005) and Brown Trout ( *Salmo trutta* [Linnaeus, 1758]) (Erős et al. 2012). Moreover, some studies have documented the importance of terrestrial invertebrates as salmonid prey and linked the provision of terrestrial insects to the influence of specific types and extents of the riparian vegetation community (Kawaguchi et al. 2003; Wipfli 1997; Baxter et al. 2005; Allan et al. 2003; Albertson et al. 2018; Roon et al. 2018; Grunblatt et al. 2019; Kotalik et al. 2023).

Riparian vegetation type, tree density, and plant community successional stage each play a role in the abundance and species assemblage of terrestrial invertebrates contributed to aquatic habitats (Wipfli 1997; Mason and Macdonald 1982; Mispagel and Rose 1978; Southwood 1961; Roegner and Johnson 2023). There is evidence that the species of riparian vegetation and canopy type can greatly influence terrestrial invertebrate abundance and may affect the amount of invertebrates that fall and become available for aquatic predators (Allan et al. 2003; Grunblatt et al. 2019; Schowalter 2017). Because land cover and plant species composition order the terrestrial invertebrate community and dictate the availability of terrestrial invertebrates (Niles and Hartman 2021), negative effects of land use can alter the linkages among habitats and communities (Foley et al. 2005) and affect the overall abundance and richness of organisms (Scharnweber et al. 2024). To that end, some studies have observed negative impacts on taxonomic and functional diversity of fish assemblages and fish size associated with changes in riparian forest cover and land use (Maracahipes‐Santos et al. 2020; Ilha et al. 2018, 2019). In a study investigating the integrity of shoreline and littoral vegetation and lacustrine damselfly habitat, Butler and de Maynadier (2008) found that littoral zones with extensive macrophyte beds were more often associated with areas of relatively undisturbed riparian habitat on both a local and lake‐wide scale, while Crosbie and Chow‐Fraser (1999) determined that reduced submergent macrophyte species richness was associated with a decreased forest and increased agricultural land use. Radomski and Goeman (2001) found that vegetative cover in littoral areas adjacent to developed shores was less abundant than along undeveloped shorelines, while Jennings et al. (2003) found that the quantity of emergent and floating vegetation and coarse wood decreased at developed sites in lakes with greater cumulative lakeshore development density.

**FIGURE 1:**
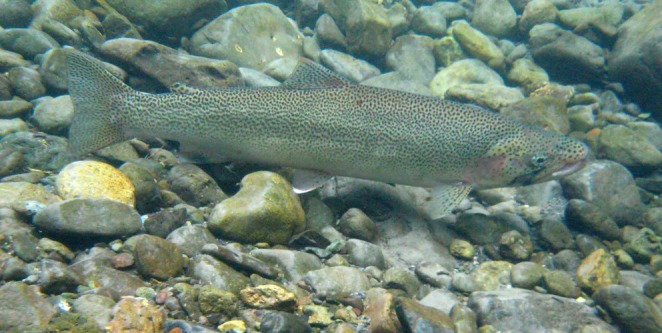
Coastal cutthroat trout in freshwater habitat on Vancouver Island, British Columbia, Canada. Photo by Mike Lough.

Coastal cutthroat trout (Figure 1), hereafter referred to as cutthroat, is an integral part of the North Pacific coast coniferous rainforest ecosystem (Trotter 2008). The species occurs along approximately 3000 km of the Pacific Northwest coast from the lower Eel River drainage of California to approximately 160 km from the coast corresponding almost exactly with that of the Pacific coast coniferous rainforest (Trotter 2008). Adfluvial cutthroat are top predators and generally reside in littoral and limnetic habitats exploiting fish and invertebrates commonly found in these zones (Beauchamp et al. 1992; Nowak and Quinn 2002). Although once widespread, the species has experienced global declines in number and distribution, and populations have become increasingly displaced from their preferred habitat because of land use including extensive forest harvesting (Precision Identification Biological Consultants 1998; Reid et al. 1998; Costello 2008; Slaney and Roberts 2005; Costello and Rubidge 2005).

Many of the low elevation riparian forests bordering the littoral zones of small lake habitats of cutthroat on Vancouver Island have been logged over the past century, some several times. In this study, we compared the diet, feeding rates, and body condition of cutthroat in lakes with lakeshore riparian zones representing a gradient of logging histories. Because the type and extent of riparian vegetation can influence the provision of terrestrial invertebrates to aquatic habitats, we hypothesized that lake riparian zones of unlogged forests would include more diverse and abundant lakeshore vegetation than second growth riparian zones. We predicted that cutthroat in these lakes would have a different diet composition and a higher intake of terrestrial invertebrates than cutthroat in lakes with logged lake riparian zones. This study provides insights into the relationship between upland terrestrial and lakeshore riparian and aquatic ecosystems and provides recommendations for future research to more clearly define the ecological connections between lakeshore riparian environments and cutthroat in small lakes on the forest land base.

## 2 Methods

### 2.1 Study Area

**FIGURE 2:**
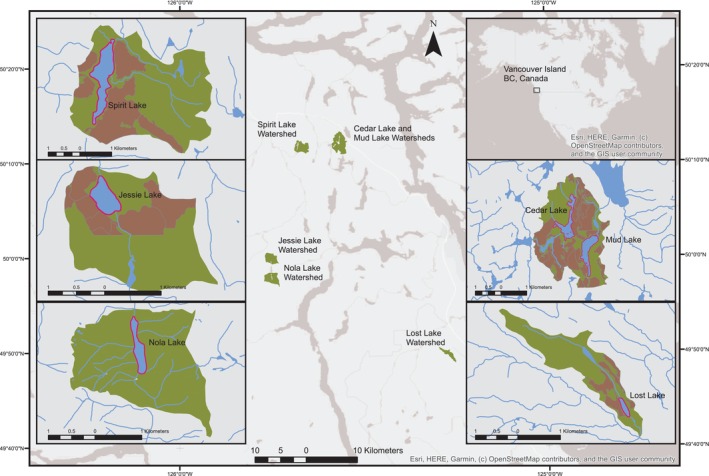
Nola, Spirit, Lost, Jessie Cedar, and Mud Lakes located in central Vancouver Island on the West Coast of British Columbia, Canada. Insert maps show 20 m riparian vegetation zone sampled in pink, harvested areas in brown and the extent of the lake watershed in dark green. All lakes are within the territories of the Mowachaht/Muchalaht, Kwakiutl, We Wai Kai, Wei Wai Kum, K'omoks, and Tlowitsis First Nations.

Our study was conducted between June and September 2016 and 2017 at six small lakes within the Leeward Island Mountains on Central Vancouver Island on the West Coast of British Columbia, Canada (Figure 2) (Demarchi 2011). The area is characterized by high, rugged mountains, rain shadows and, at times, heavy precipitation because of moist Pacific air moving over the western side of Vancouver Island. Summers can be hot and dry and although winters are generally wet and mild there can be infrequent cold periods with snow in lowland areas. The mean annual temperature of the area is approximately 9°C, with a summer mean of 14°C and a winter mean of 3.5°C. Mean annual precipitation ranges from 800 mm at lower elevations to 2500 mm at higher elevations (Nature Conservancy of Canada, n.d.). In addition to many small lakes, there are several large lakes (> 1000 ha) and rivers, and numerous shorter rivers originating in the lowlands. The region is among the most biologically diverse of any area of the province and characteristic wildlife include black‐tail deer ( *Odocoileus hemionus columbianus* [Richardson 1829]), cougar ( *Puma concolor* [Linnaeus, 1771]), gray wolf ( *Canis lupus* [Linnaeus, 1758]), American black bear ( *Ursus americanus* [Pallas, 1780]), raccoon ( *Procyon lotor* [Linnaeus, 1758]), sea otter ( *Enhydra lutris* [Linnaeus, 1758]), and river otter ( *Lontra canadensis* [Schreber, 1777]) (Nature Conservancy of Canada, n.d.). Many species of shorebird, seabirds and waterfowl occur throughout the year and the region also supports coho, chinook ( *Oncorhynchus tshawytscha* [Walbaum, 1792]), pink ( *Oncorhynchus gorbuscha* [Walbaum, 1792]), chum ( *Oncorhynchus keta* [Walbaum, 1792]), and sockeye/kokanee ( *Oncorhynchus nerka* [Walbaum, 1792]) salmon, steelhead and rainbow trout ( *Oncorhynchus mykiss* [Walbaum, 1792]), Coastal cutthroat trout and Dolly Varden char.

Forest cover throughout the region is characterized by stands of Douglas‐fir ( *Pseudotsuga menziesii* [Mirb, Franco 1950]), western hemlock ( *Tsuga heterophylla* [Raf, Sarg. 1898]), and western redcedar ( *Thuja plicata* [Donn 1811]), with lesser amounts of grand fir ( *Abies grandis* [Douglas ex D. Don Lindl, 1833]), and an understory of salal ( *Gaultheria shallon* [Pursh 1813]), Oregon grape ( *Mahonia nervosa* [Pursh, Nutt. 1818]) and mosses. Common riparian and early successional forest species include red alder ( *Alnus rubra* [Bong 1832]), black cottonwood ( *Populus balsamifera ssp. trichocarpa* [Torr & A. Gray, Brayshaw 1965]), and bigleaf maple ( *Acer macrophyllum* [Pursh 1813]) with a diverse understory. Many of these forests are in stages of second growth because of an extensive history of logging over the past century (Perrin and Blyth 1998). Resource extraction (logging as well as mining) has resulted in many forest access roads throughout the area (Demarchi 2011).

### 2.2 Study Design and Lake Characteristics

Study lakes were identified from several potential watersheds and were included in the study if they had wild cutthroat, < 1 km^2^ surface area, < 10 m mean depth, ≤ 500 m elevation, a suitable boat launch, accessible by road, and no fish stocking within the previous decade.

Although water quality data are sparse, existing records document low dissolved solids concentrations resulting in oligotrophic lakes (i.e., Total Dissolved Solids [TDS] < 75 ppm, with Total Phosphorus concentrations mostly < 0.010 mg/L) (Table A1) resulting in low standing crops of plankton, bottom fauna, and fish (Perrin and Blyth 1998; Northcote and Larkin 1956). This is expected because of dilution from heavy rainfall combined with low weathering rates of hard granitic bedrock characteristic of the region (Perrin and Blyth 1998). Additionally, the low nutrient loading rates to the aquatic systems in this area are enhanced by nutrient retention in second growth forests which contributes to oligotrophication of regional lakes and streams (Perrin and Blyth 1998).

Lake watersheds are within the very dry maritime and the adjacent moist maritime subzones of the Coastal Western Hemlock (CWH) Biogeoclimatic Ecosystem Classification (BEC) zone, and higher elevation lake watersheds (Nola and Jessie Lakes) also include a small portion of forest within the Mountain Hemlock (MH) zone (Green 1994). The lake riparian vegetation at Nola and Spirit Lakes includes old growth forest (> 250 years); however, the Nola Lake watershed is comprised entirely of old forest while just over 30% of the Spirit Lake watershed has been harvested. Approximately half of the riparian is < 80 years old at Lost Lake, with approximately 17% of the watershed harvested (Table A2) (Government of British Columbia 2025). Roughly 75% of the lake riparian at Jessie Lake is comprised of trees < 80 years old, with approximately 33% of the watershed harvested. Approximately half of the riparian at Cedar Lake is < 80 years old, with roughly 38% of the watershed harvested. Over 75% of the riparian zone within 20 m of shore at Mud Lake is comprised of forest > 80 years old, while 41% of the watershed has been logged. Harvesting within 20 m of the lakeshore has occurred at all but Nola and Spirit Lakes. Forest harvest began at Spirit and Lost Lakes in 2014 and 2010, respectively, and at Jessie Lake in 1979. Harvesting at Cedar and Mud lakes began in 1947.

### 2.3 Field Methods

Lake riparian forests were not homogeneous. We identified the three dominant lakeshore site types according to forest age and environmental characteristics (e.g., substrate, terrain, slope, disturbance) at each lake using a combination of satellite imagery and field observation. Vegetation sampling was conducted in August and September of 2016 and 2017. We sampled 2 × 2 m plots along each of three randomly located transects placed perpendicular to the lakeshore within each of the three site types. Aquatic plots (plots 1 and 2) extended from the lakeshore into the lake littoral zone, while the upland five plots extended from the lakeshore into the riparian and upland terrestrial zones. We visually estimated percent cover of individual species for each vertical vegetative layer and the percent decaying wood ≥ 10 cm in diameter within each plot to describe the riparian vegetation community and habitat complexity. Vertical layers in the aquatic plots were categorized as overhanging (growing above lake surface), floating (floating on the lake surface), or submerged (below lake surface), while vertical layers of terrestrial plots were characterized by height (> 10 m [overstory], 2–10 m [shrub], < 2 m [understory]). Vegetation species were grouped into five categories (evergreen tree, deciduous tree, evergreen shrub, deciduous shrub, herb) for data analyses.

Surface water temperature, dissolved oxygen, and conductivity measurements were taken monthly along the littoral zones. Water temperatures and dissolved oxygen readings were taken using a YSI Pro1020 Dissolved Oxygen meter, while conductivity was measured using a YSI Pro30 Conductivity handheld meter. Water quality data are provided in Table A1.

Fish were sampled by fly fishing monthly from June until September 2017. Fly fishing is a specialized angling technique that uses artificial lures made from natural materials such as feathers, fur, and thread. Lures are designed to imitate both the look and behavior of native invertebrates. Angling with a rod and reel has been used for acquiring fish for scientific purposes, including radiotelemetry and genetic and population structure and abundance studies (West et al. 1992; King and Pate 1992; Murphy and Willis 1996). We chose fly fishing because the lures imitate natural invertebrate prey and are not ingested, so they can be removed easily so that fish and stomach contents can be sampled quickly, thereby minimizing fish mortality.

A total of 196 cutthroat were caught by flyfishing by boat in the littoral zones for 2 h every 4 weeks at each lake (average Catch Per Unit Effort [CPUE] = 3.9). Fish were anesthetized using Tricaine‐S (MS‐222), weighed, measured, and sampled for scales. We used the lavage method (Culp et al. 1988; Haley 1998; Meehan and Miller 1978) to collect stomach contents from live cutthroat. Stomachs were flushed three times using a plastic syringe attached to a small plastic tube. Stomach contents were emptied into a collecting tray, then poured into sample jars and preserved with 95% denatured ethanol. Fish were revived in fresh lake water and returned to the lake.

Stomach contents from 196 cutthroat (Nola Lake—58, Spirit Lake—16, Lost Lake—53, Jessie Lake—19, Cedar Lake—15, Mud Lake—35) were sent to invertebrate taxonomists for analyses. Identifications were made at the genus/species level for all insects. Noninsects were identified to genus/species where possible and to a minimum of family level. If specimens could not be identified to the specified level, they were grouped into terrestrial or aquatic “other” categories. Cutthroat were aged by mounting scales on glass slides, photographing each scale, annotating, and counting annuli on each photograph. Results of fish analyses are provided by fish year class and in some cases grouped according to juvenile (1YO, 2YO, 3YO) or adult (4YO, 5YO, 6YO) categories. Age category groupings are based on available literature which suggests an average age range of 2 YO–4 YO for freshwater and anadromous cutthroat to reach sexual maturity (Trotter 2008; Scott and Crossman 1973). Four cutthroat from Nola, two from Spirit, and one each from Jessie and Cedar Lakes were not aged because of regenerated scales. These fish are not included in data summaries.

### 2.4 Data Analysis

#### 2.4.1 Riparian Vegetation and Decaying Wood

We used forest cover spatial layers available from the Ministry of Forests (Province of BC 2023) to identify harvest block sizes and ages for each of our study lakes. We used Google Earth Pro to estimate the percentage of each site type, then weighted each site type to characterize the vegetation and reflect the biophysical character of the lakeshore riparian forest at each lake. We determined the gradient of harvested riparian vegetation by calculating the percentage of logged area within 20 m of the lakeshore. All results are presented in order of least to highest percentage of logged area within 20 m of the lakeshore.

We used a one‐way Analysis of Variance (ANOVA) (R Core Team 2025) to determine if there were differences in the percentages of vegetative cover by vertical layer among lakes. Percent decaying wood and vegetation data were summarized to the lake level for analyses and comparisons to fish diet, feeding rates, and body condition using Pearson's correlation coefficients. To summarize to the lake level, we first computed mean percent cover for aquatic and terrestrial plots and then combined the results for the three site types using a weighted average to represent the relative contribution to percent cover as:

$${\bar{x}}_{w}=\frac{\sum_{i=1}^{n}w_{i}x_{i}}{\sum_{i=1}^{n}w_{i}}$$

where ${\bar{x}}_{w}$ = weighted average cover, *n* = 3 = number of site types to be averaged, $w_{i}$ = weight characterizing proportional representation of each site type, $x_{i}$ = average percent cover for each site type.

#### 2.4.2 Cutthroat Size, Feeding Rate, and Body Condition

The complete sample of fish was not balanced across age classes at each lake. To minimize the impact of these age class differences among lakes, we used a Monte Carlo sampling approach that randomly subsampled each lake's age classes with a balanced sample based on the smallest (non‐zero) sample size for that lake. Some lakes may still have an age class(es) without samples, but the overall imbalance is largely addressed without losing data. We report the average of mean fish attributes, percentages, statistics and *p*‐values (when appropriate) based on 10,000 random balanced samples via the R package “sampling” version 2.10 (Tillé and Matei 2021).

We used a one‐way ANOVA to determine if there were differences in the mean fork lengths, feeding rates, and condition factors among juvenile (1 YO, 2YO, 3YO) and adult (4YO and older) age classes. Average *p*‐values are based on 10,000 *F*‐tests after sampling the age classes.

We compared the proportion of terrestrial to aquatic invertebrates in the diets of juvenile and adult cutthroat at study lakes. We calculated the allometrically‐corrected feeding rate (FR) (i.e., proportion of daily consumption) for every fish to maximum possible consumption ($C_{\max}$) based on physiology observed in each fish (Eby et al. 1995) as:

$$\mathrm{FR}=D/C_{\max}$$

where $C_{\max}=0.628\;W^{-0.3}$, and W = individual fish wet weight in grams.

We used a modified Eggers model provided in Francis and Schindler (2009) to calculate daily ration (D). Daily ration is the daily rate of total and aquatic versus terrestrial consumption for each fish, and has units of grams prey wet mass per grams fish wet mass per day:

$$D=24\cdot F\cdot R_{e}$$

where $R_{e}$ = gastric evacuation rate (h^−1^) = $0.049\;\exp\left(0.072\;T-0.060\;\ln\left[\mathrm{PS}\right]\right)$, T = temperature in °C, PS = mean prey wet weight in grams, F = gut fullness.

Gut fullness (F) was calculated according to Principe et al. (2007) as:

F=G/W

where G = gut weight of aquatic, terrestrial, or both sources of food in grams.

We calculated the condition factor (K) (Williams and Schneider 2000) to compare the body condition of each cutthroat sampled at each lake as:

$$K=100W/L^{3}$$

where L is the fish fork length in cm.

#### 2.4.3 Relationships Among Cutthroat Diet, Feeding Rate, and Body Condition and Riparian Vegetation

We computed Pearson's correlation coefficients to determine if there were relationships among feeding rate, condition factor, intake of terrestrial, and aquatic invertebrates and mean percent cover of riparian vegetation by vertical layer and decaying wood. Additionally, we computed Pearson's correlation coefficients to determine if there were relationships between the mean percentages of aquatic and terrestrial vegetative layers.

We used classical metric multidimensional scaling (MDS) to graphically display the dissimilarity among lakes based on vegetation and decaying wood cover (four aquatic layers [three vegetation and one decaying wood] + four terrestrial layers [three vegetation and one decaying wood]). Similarly, we used MDS to display the dissimilarity among the lakes with respect to mean fish characteristics (wet mass of terrestrial invertebrates, wet mass of aquatic invertebrates, feeding rate, and condition factor) for both juveniles and adults. (Gower 1966). All MDS figures used the cmdscale function and depict underlying Euclidean distances on the original (raw) scale. To display the relationship between vegetation cover or fish characteristics and the resulting MDS axes, we used the envfit function from the R package “vegan” (Oksanen et al. 2025). Stress (lack‐of‐fit) for each figure was determined using the R package “smacof” (Mair et al. 2022).

## 3 Results

### 3.1 Riparian Vegetation and Decaying Wood

**FIGURE 3:**
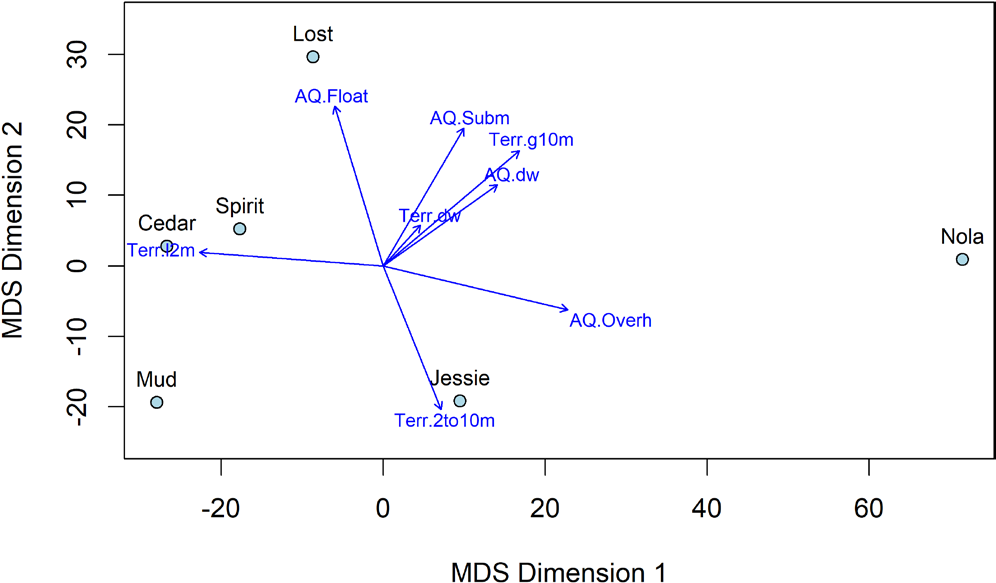
Multidimensional scaling graph showing dissimilarities among lakes with respect to aquatic and terrestrial vegetation within the riparian habitat of Nola, Spirit, Lost, Jessie, Cedar, and Mud Lakes on Vancouver Island, BC. Axes reflect distances based on weighted average cover of overhanging, floating and submerged (aquatic) and overstory, shrub and understory (terrestrial) plots, along with decaying wood ≥ 10 cm diameter. Stress = 0.063. Relationships of vegetation attributes with MDS axes are displayed as blue arrows (AQ.Float = aquatic floating cover, AQ.Subm = aquatic submerged cover, AQ.Overh = aquatic overhanging cover, AQ.dw = aquatic decaying wood, Terr.l2m = terrestrial understory cover Terr.2to10m = terrestrial shrub cover, Terr.g10m = terrestrial overstory cover, Terr.dw = terrestrial decaying wood).

Study lakes were dissimilar with respect to the percentage of vegetative and decaying wood cover provided by aquatic and terrestrial strata, with cover at Nola, and to a lesser extent at Jessie Lakes, differing from the remaining four lakes (Figure 3). Clear differences regarding the vegetative percent and composition within strata were evident among lakes. For example, Nola Lake has substantially more (nearing 90%) vegetative cover in the terrestrial overstory (> 10 m), all of which was provided by evergreen trees compared to between ~30% and ~60% at remaining lakes (Table A3). Additionally, Nola Lake has considerably more cover (75%) overhanging the aquatic (littoral) zone, with most of this cover provided by evergreen trees and deciduous shrubs. In contrast, the average percentage cover provided by overhanging vegetation at Jessie Lake was almost half that (42%), and at remaining lakes varied from 2% (Cedar Lake) to 22% (Lost Lake). We also noted a large difference in the percentage of terrestrial understory vegetation among lakes, with this stratum at Nola Lake comprising roughly half that of remaining lakes.

**FIGURE 4:**
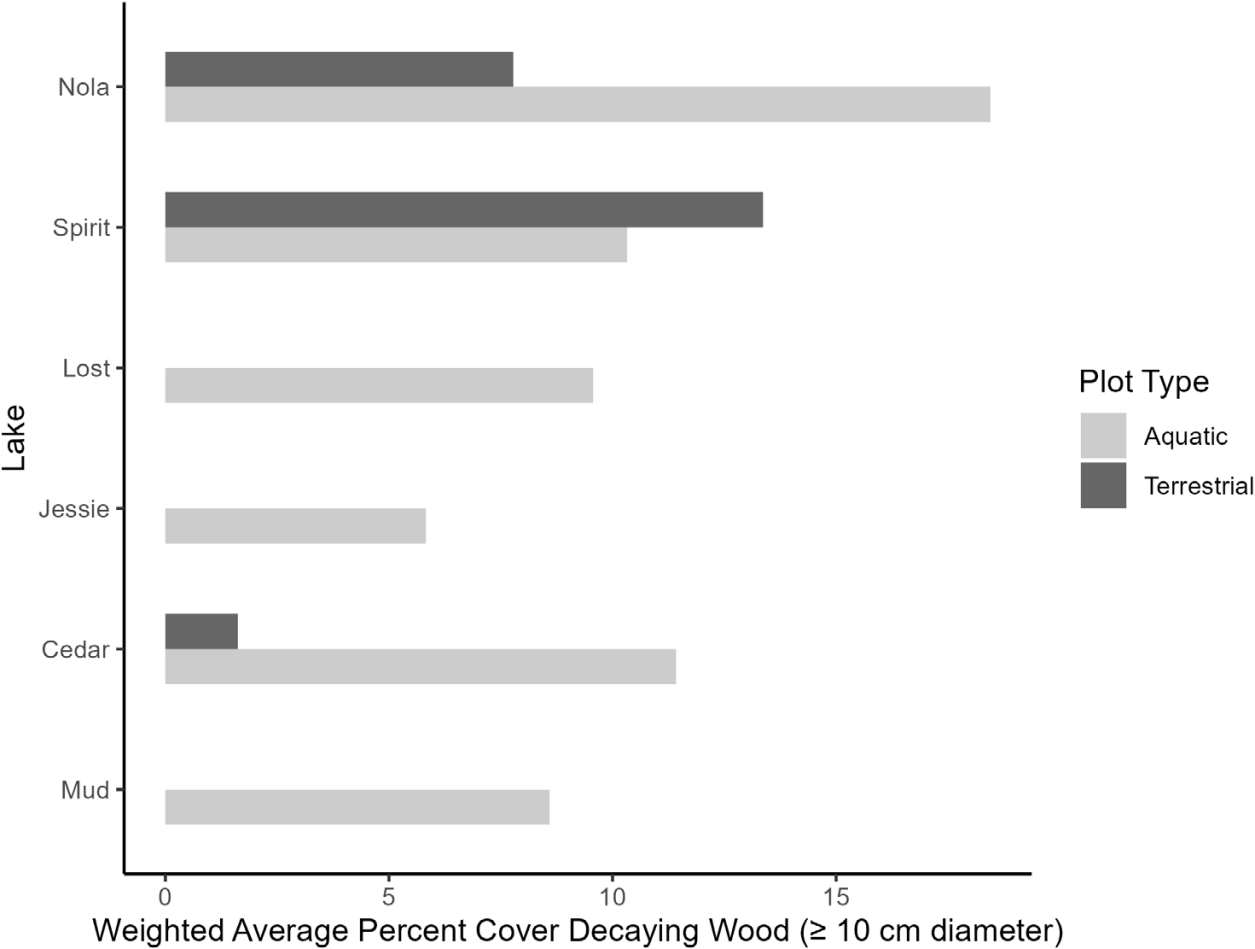
Weighted mean percentage and distribution of large (≥ 10 cm diameter) decaying wood in aquatic and terrestrial plots at Nola, Spirit, Lost, Jessie, Cedar, and Mud Lakes on Vancouver Island, British Columbia, Canada.

We found a strong, positive relationship (*r* = 0.81, *p* = 0.0489) between the weighted average percent cover of terrestrial overstory and aquatic overhanging vegetation (Table A4). Similarly, a strong, positive relationship (*r* = 0.76, *p* = 0.0786) between the percentage of submerged vegetation and the percentage of overstory terrestrial vegetation also exists. Again, Nola Lake had a higher percentage of submerged vegetation (33%) compared to that at remaining lakes where the average submerged cover varied from < 1% (Jessie and Mud Lakes) to 22% (Lost Lake). We also noted a strong, negative relationship (*r* = −0.88, *p* = 0.0216) between the percentage of terrestrial understory and the percentage of aquatic overhanging vegetation. Finally, we observed a considerable difference in the percentage of decaying wood among study lakes (Figure 4). For example, while we observed decaying wood in the aquatic plots at all lakes, we only noted large wood in the terrestrial plots at Nola, Spirit and, to a lesser extent, Cedar Lakes.

### 3.2 Cutthroat Size, Feeding Rate, and Body Condition

**FIGURE 5:**
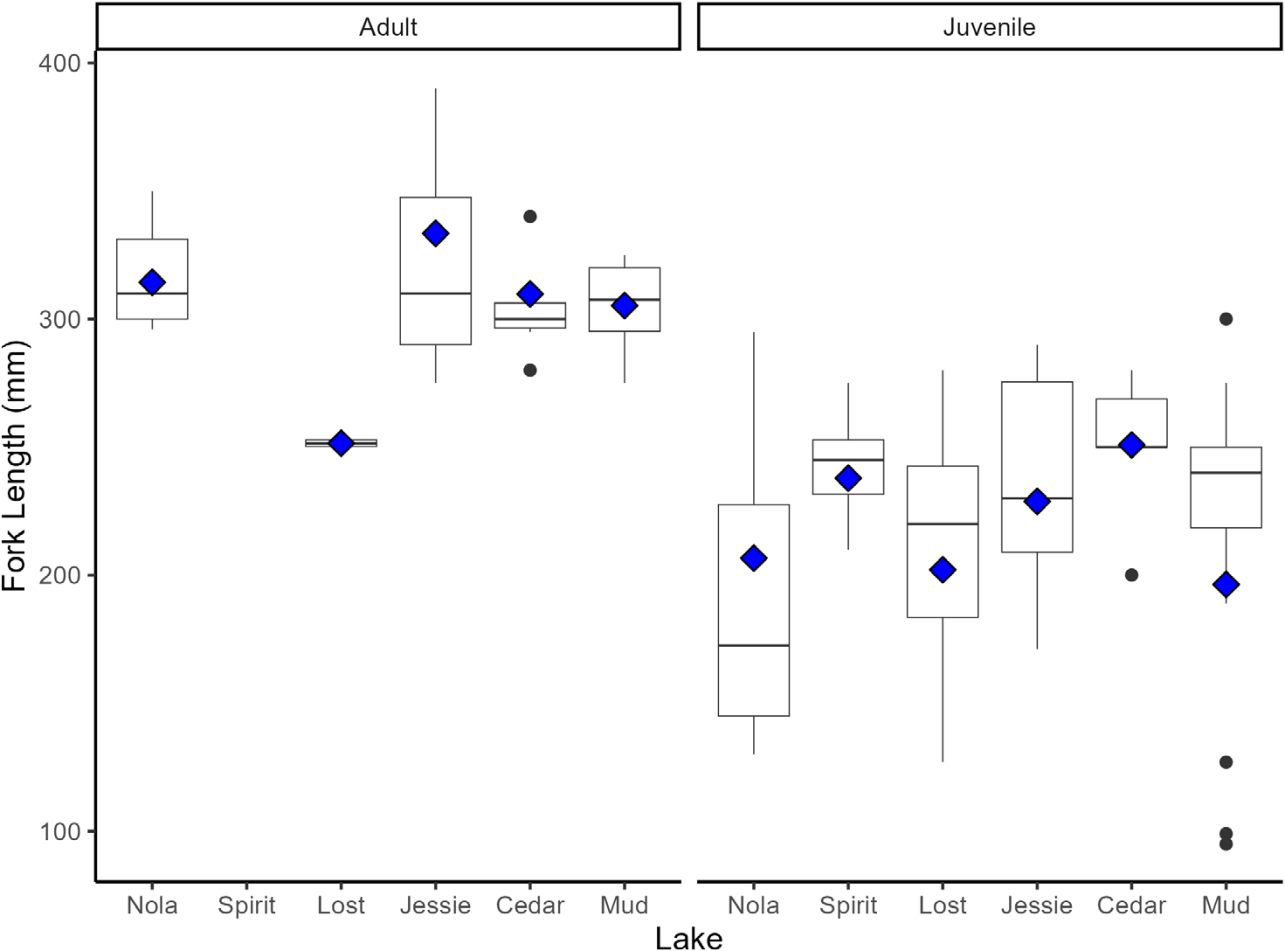
Distributions of Fork length (mm) of adult (4YO, 5YO, 6YO) and juvenile (1YO, 2YO, 3YO) cutthroat captured June–September 2017 at Nola, Spirit, Lost, Jessie, Cedar, and Mud Lakes on Vancouver Island, British Columbia, Canada. Diamonds within boxes are means based on Monte Carlo balanced samples (see Section 2).

We captured cutthroat ranging from 1YO to 6YO (Figure 5, Table A5). While we found no difference in the fork lengths of juvenile (1YO, 2YO, 3YO) cutthroat among lakes (Fork Length Juv: df = 5, 71 avg. *p* = 0.1177), we did find a significant difference in the length of adult (4YO+) cutthroat among lakes (Fork Length Adult: df = 4, 15 avg. *p* = 0.0247).

**FIGURE 6:**
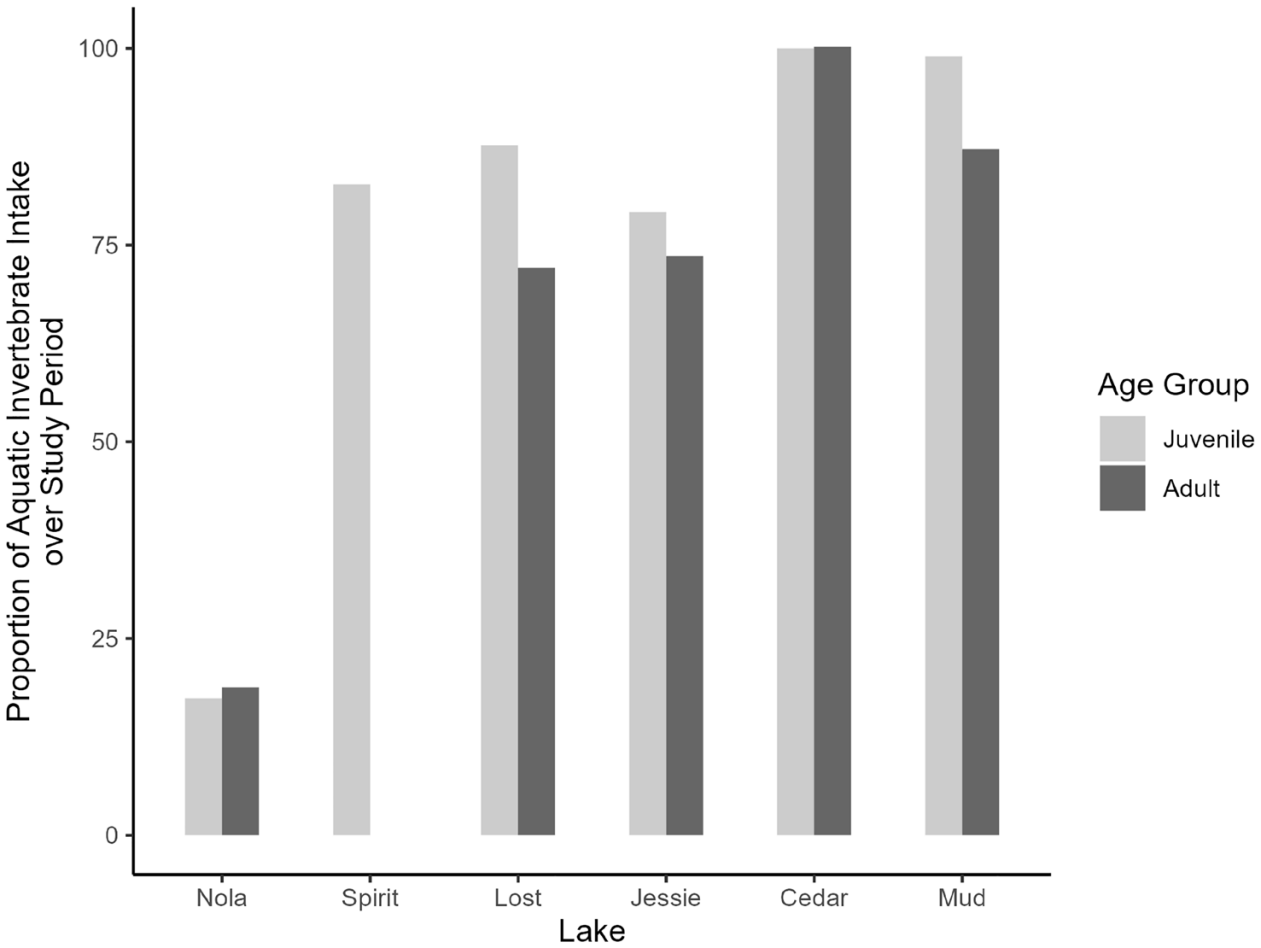
Percentage intake of aquatic invertebrates comprising the diets of juvenile (1YO, 2YO, 3YO) and adult (4YO, 5YO, 6YO) cutthroat June–September 2017 at Nola, Spirit, Lost, Jessie, Cedar, and Mud Lakes on Vancouver Island, British Columbia, Canada.

The intake of terrestrial versus aquatic prey by both juvenile and adult cutthroat was very different at Nola Lake compared with that of cutthroat sampled at the remaining lakes (Figure 6). In all but Nola Lake, the proportion of aquatic prey was much higher than the proportion of terrestrial prey, and in some cases, aquatic prey made up the entire diet. At Nola Lake, however, the proportion of terrestrial invertebrates was three times that of aquatic invertebrates. Specifically, members of the Order Hymenoptera (Linnaeus, 1758) were among the most common and contributed the greatest biomass (by wet weight) prey items in the diets of juvenile cutthroat, while other unidentified terrestrial invertebrates made up almost 100% of the diet of adult cutthroat at Nola Lake (Table A6). Although terrestrial invertebrates were present in the stomachs of juvenile cutthroat at Spirit Lake, members of the Order Ephemeroptera (Hyatt and Arms, 1890) were both more common and contributed more biomass to the diets of juvenile cutthroat at this lake. Terrestrial prey items were present in the diets of juvenile and adult cutthroat at Lost and Jessie Lakes; however, by far the most common prey items and those that contributed the most biomass to the diets of fish at Cedar and Mud Lakes were members of the superorder *Cladocera* (Latreille, 1829) and the class Copepoda (Milne‐Edwards, 1840).

**FIGURE 7:**
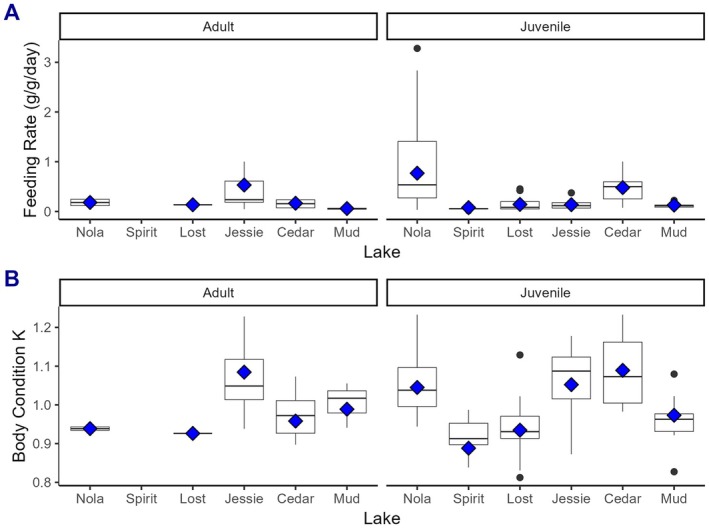
Distribution of feeding rate (A) and body condition (B) of adult (4YO, 5YO, 6YO) and juvenile (1YO, 2YO, 3YO) cutthroat captured June–September 2017 at Nola, Spirit, Lost, Jessie, Cedar, and Mud Lakes on Vancouver Island, BC. Diamonds within boxes are means based on Monte Carlo balanced samples (see Section 2).

We found a significant difference in both the feeding rates (Feeding Rate Juv: df = 5, 60 ave. *p* = 0.0029) and body condition of juvenile cutthroat among lakes (Body Condition Juv: df = 5, 60 ave. *p* = 0.000037) (Figure 7). In contrast, neither the feeding rate (Feeding Rate Adult: df = 4, 7 ave. *p* = 0.4072) nor body condition of adult age classes differed among lakes (Body Condition Adult: df = 4, 7 ave. *p* = 0.2521).

**FIGURE 8:**
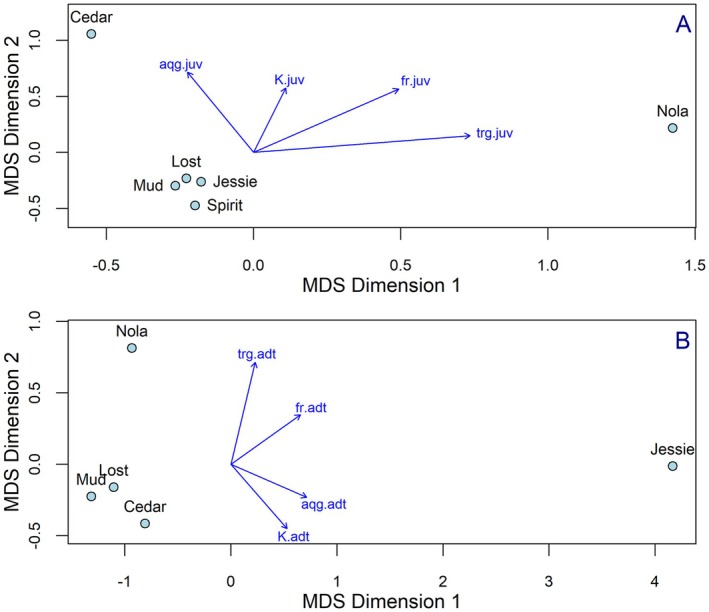
Multidimensional scaling graph for dissimilarities among lakes for juvenile (1YO, 2YO, 3YO) (A) and adult (4YO+) (B) fish with respect to diet, feeding rate and body condition and wet mass of aquatic and terrestrial riparian vegetation. Stress = 0.012 (A) and 0.001 (B). Relationships of four attributes with MDS axes are displayed as blue arrows (aqg.juv or aqg.adt = aquatic intake (wet mass), trg.juv or trg.adt = terrestrial intake (wet mass), fr.juv or fr.adt = feeding rate, K.juv or K.adt = body condition). Cutthroat were captured June–September 2017 at Nola, Spirit, Lost, Jessie, Cedar and Mud Lakes on Vancouver Island, British Columbia, Canada.

Our MDS ordination suggests that juvenile (1YO, 2YO, 3YO) cutthroat sampled from Nola and Cedar Lakes are unique with respect to diet, feeding rates, and body condition compared to juvenile cutthroat sampled from all other study lakes (Figure 8). Similarly, our analysis suggests that adult (4YO+) cutthroat from Nola and Jessie Lakes are different from adult cutthroat sampled from the remaining study lakes.

### 3.3 Relationships Among Cutthroat Diet, Feeding Rate, and Body Condition and Riparian Vegetation

**FIGURE 9:**
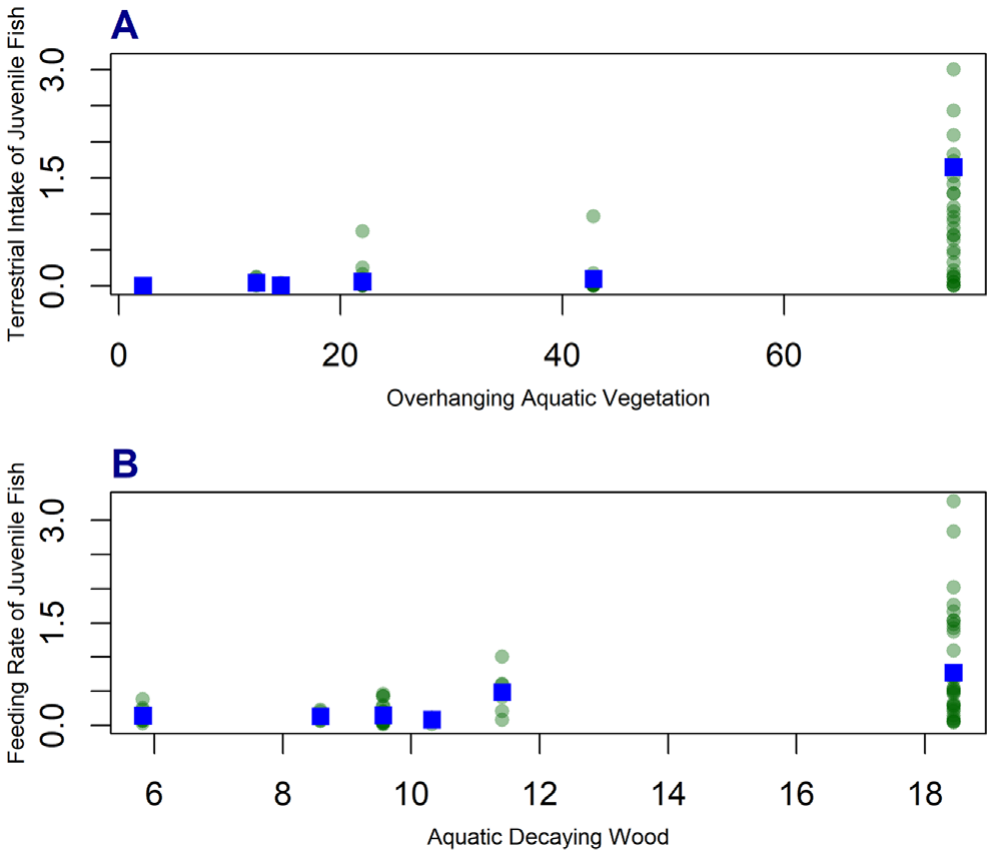
Scatter plots showing the relationship between the intake (wet mass) of terrestrial prey by juvenile (1YO, 2YO, 3YO) cutthroat and average percentage overhanging aquatic vegetation (A), feeding rate of juvenile cutthroat and average percentage aquatic decaying wood (B). Fish observations are shown as green points for visualization only. Balanced age‐class corrected means are shown as blue squares. Note that in plot A, five fish observations are not shown (*y* > 4) at *x* = 75.3.

We found several relationships when we compared cutthroat diets to the percentage of vegetative cover provided by individual vegetation strata and decaying wood. For example, we found positive and, in some cases, significant correlations between the intake of terrestrial invertebrates by juvenile and adult cutthroat and the percentage of overhanging and submerged vegetation and decaying wood within the littoral zone (Figure 9, Table A7). Additionally, we found strong, positive relationships between the feeding rates of juvenile cutthroat and the percentage of submerged vegetation and decaying wood within the littoral environment.

## 4 Discussion

Riparian vegetation overhanging and submerged along the lakeshore provides an important connection and a direct supply of terrestrial invertebrates to littoral habitats, and our results suggest that diminished amounts of that vegetation may have implications for foraging cutthroat. Our study found that cutthroat of all ages fed almost exclusively on terrestrial invertebrates at a lake with extensive overhanging riparian vegetation compared to cutthroat that fed on aquatic invertebrates in lakes with limited overhanging vegetation. While overhanging vegetation is an essential element in the provision of terrestrial prey to foraging cutthroat (Mason and Macdonald 1982; Romero et al. 2005; Sanders et al. 2024) we found that for juvenile cutthroat in particular, submerged vegetation has a positive effect on the total intake and feeding rate on terrestrial diet items. We predicted that cutthroat in lakes surrounded by unlogged riparian areas would have a higher intake of terrestrial invertebrates than cutthroat in lakes with logged riparian zones. In our study, it is possible that not only were terrestrial invertebrates more abundant because of the presence of overhanging vegetation, but also that trout were selecting terrestrial items over aquatic diet items that were also available. However, our results are consistent with those of Cadwallader et al. (1980) who determined that terrestrial invertebrates formed a substantial part of the diets of fish sampled from sites in lakes surrounded by overhanging vegetation and were much less common in the diets of fish taken from sites with little overhanging vegetation. That same study also found that the terrestrial food source was eliminated from fish diets when the overhanging vegetation was removed, which supports the findings of other studies showing the greater the terrestrial prey influx, the greater its contribution to the diets of fish (Nakano and Murakami 2001; Sánchez‐Hernández and Cobo 2016).

Our results indicate that along with the importance of submerged vegetation, higher feeding rates, and higher intakes of terrestrial invertebrates by juvenile cutthroat are also positively related to the percentage of decaying wood along the lakeshore. Because it affects the flow of organic matter from terrestrial ecosystems into surface waters, decaying wood is a critical component of the littoral ecosystem and an important link between forested and aquatic ecosystems (Bormann and Likens 1979; Franklin 1981; Christensen et al. 1996). Some studies (Dolloff and Warren 2003; Gurnell et al. 2005; Spänhoff et al. 2000) have found that large wood has major effects on aquatic biota, including for feeding fish. Forest harvest can initially increase coarse wood inputs into lake habitats by introducing large amounts of slash and other debris (Spies et al. 2018) but reduces wood inputs over longer time periods because of the loss of riparian trees (Harmon et al. 1986; Murphy and Koski 1989; Bilby and Ward 1991). Except for Nola and Spirit Lakes, we documented very little, and in some cases, no decaying wood in the littoral and riparian zones of lakes that have had some harvest in their basins. The fact that the most common and dominant food items for all age categories of cutthroat at Nola Lake were Hymenoptera and Dipterans, both of which are saproxylic to some extent (de Groot et al. 2016) provides additional support for the positive association we found between the intake of terrestrial prey items and riparian decaying wood. It is possible that the higher feeding rates of juvenile cutthroat were also related to other factors, including lower water temperatures, such as we observed at Nola Lake (Table A1) and which would affect both fish physiology and behavior, including feeding activity (Brett 1952; Volkoff and Rønnestad 2020; Edmundson and Mazumder 2001). Foraging tactics depend on many factors, including the direct benefit from available resources, inter‐ and intraspecific competition, aggression and predation, and changes in food availability (Huey and Pianka 1981; Denoël 2004). However, structurally more complex areas with higher percentages of aquatic macrophytes and large wood are important for providing a greater abundance, biomass, and diversity of macroinvertebrates, shaping foraging behavior and increasing prey intake (Nowak and Quinn 2002; Edmundson and Mazumder 2001; Pardue and Webb 1985; Beckett et al. 1992; Rennie and Jackson 2005; Blindow et al. 1993; Harding 2004). Structurally complex and highly vegetated littoral zones such as that at Nola Lake provide protective cover and feeding sites rich in terrestrial prey, allowing juvenile cutthroat that preferentially forage among these areas the opportunity to encounter sufficient and preferred terrestrial prey with minimal energy expense and exposure to threats (Trotter 2008; Fausch 1984; Hughes and Dill 1990; Northcote and Atagi 1997).

We identified higher feeding rates by juvenile cutthroat in Nola Lake and expected that higher body condition of these age classes would also be evident, particularly given the higher prey energies of species such as Hymenoptera (Gray 2005) compared to that of aquatic prey including Copepoda (Koehler et al. 2006) ingested by juvenile cutthroat at lakes with less highly vegetated and complex littoral ecosystems. Differences in juvenile body condition among study lakes were not clear, however, and we were also unable to identify differences in the feeding rate or body condition of adult age classes among lakes. Like many fish species, salmonids typically exhibit indeterminate growth across their life span and, as a result, they require a diversity of habitats and have been observed to alter their habitat preferences as they grow (Bjornn and Reiser 1991; Northcote 1997; Quinn 2018; Rosenfeld and Taylor 2009; Gross et al. 1988; Hallbert and Keeley 2024; Li et al. 2016). Consequently, our littoral‐zone focused sampling likely did not allow for adequate sampling of older age classes of cutthroat and may not have provided a full picture of the positive effects of feeding rate on adult size and body condition. Moreover, salmonids exhibit large variations in body size and their growth rates are controlled by many factors in addition to size and energy intake, including density‐dependent (i.e., the compensatory manner in which populations respond to changes in abundance) and density‐independent aspects (i.e., environmental factors) such as temperature (Grant et al. 2017; Elliott 1994; Huntsman et al. 2021) and primary productivity (Karlsson and Byström 2005; Norman et al. 2022) which likely is limited in the oligotrophic lakes we sampled.

For many jurisdictions, the underlying objective of leaving riparian buffers around aquatic habitats is to isolate upland activities from terrestrial nearshore and aquatic areas (Lee et al. 2004). However, the long‐term sustainability of riparian management zones depends upon the maintenance of desirable ecosystem conditions within those riparian buffers (Zenner et al. 2012). Our results suggest that the differences in the amounts of lakeshore vegetative cover among harvested versus unharvested lakeshore riparian areas can be explained by a strong, positive relationship with the percentage of overstory in the adjacent terrestrial riparian zone. Our findings are in keeping with the results of several studies (Zenner et al. 2012; Brosofske et al. 1997; Dignan and Bren 2003; Dieterich et al. 2006) which have shown that partial overstory removal can have a significant impact on understory vegetation and function of forested riparian areas at least in the short term.

The nature of the forest ecosystem, along with a variety of local and regional factors, determines how far the edge influence extends into the intact forest (Harper et al. 2005). The response of vegetation within the interior forest can result directly from wind and microclimate effects of forest removal or can arise as a secondary response from modifications to resource availability (light, water, nutrients) and can result in changes in understory and/or herbaceous community structure and species (Zenner et al. 2012; Wales 1972; Laurance et al. 2002; Oosterhoorn and Kappelle 2000). Indirect effects of land management in a watershed can effectively narrow the riparian buffer zone adjacent to aquatic habitats because of the response of the interior forest to the creation of edges at the harvest margin (Zenner et al. 2012; Brosofske et al. 1997; Dignan and Bren 2003; Dieterich et al. 2006; Chen et al. 1995). Additionally, although forest vegetation responses to edge effects are usually a function of microclimate condition, changes to the amount of downed coarse wood are also a primary response to a created edge (Kremsater and Bunnell 1999; Harper and Macdonald 2001). Our findings could be a result of a response to different light conditions at harvested versus unharvested lakeshores. Given that compared to unmanaged riparian areas, managed riparian forests have been found to have significantly higher average light levels with associated increases in more shade‐intolerant species in the understory, especially within 12 m of the edge of a stream (Dieterich et al. 2006). However, our project was limited by the number of study sites that fit our experimental design of small lakes with riparian areas representing a gradient of forest harvest. Consequently, we recommend that future studies examine the connections between forest harvest and the extent, composition, and response of variables that influence the complexity of littoral vegetation at lakes within Pacific coast watersheds.

In addition to limited study sites, we also chose to capture cutthroat by fly fishing on the littoral zone and recommend that future studies consider also using minnow traps and netting to capture more fish of each age class in the limnetic zone of the lake. Our sampling occurred in the summer and prey utilization varies according to season (Kotalik et al. 2023; Li et al. 2016; Burbank et al. 2022). Future research could account for the temporal variability of prey availability and use by trout. Finally, although our data suggest that the extent of vegetation overhanging and submerged along the littoral zone may be responsible for high intakes of terrestrial invertebrates and higher feeding rates of juvenile foraging cutthroat, there are likely other factors contributing to these observations that future studies should consider such as fish behavior and competition, the length of the growing season at higher elevation lakes, the number of tributary inputs, groundwater upwelling, and lake temperatures.

## 5 Conclusion

Despite the limitations of our study, we identified an association between cutthroat diet and condition, and vegetation and decaying wood, suggesting that these factors may, at least in part, affect the feeding ecology and condition of adfluvial cutthroat trout. We found that the extent of these elements is positively related to the percentage of overstory vegetation within the lakeshore riparian zone. Our study also identified differences in the percentage and composition of aquatic and riparian terrestrial vegetative strata between a lake within an unharvested watershed and lakes with a gradient of watershed harvest, including, in some cases, within 20 m of shore. In streams, the loss of processes that contribute to physical habitat complexity is often caused by degradation of riparian habitat (Hallbert and Keeley 2023). A reduction in habitat quality can affect the survivorship, growth, or reproductive capacity of individuals and may lead to population decline and extirpation (Hallbert and Keeley 2023; Brewster et al. 2018; McNeil et al. 2020). Moreover, there is a potential to affect fish if the creation of a harvest edge compromises the amount of light and ultimately terrestrial invertebrate input from riparian to aquatic habitats (Erős et al. 2012; Nakano and Murakami 2001; Gregory et al. 1991). Although connections between aquatic and terrestrial habitats are becoming clearer, ecologists are still working towards understanding trophic relationships among species and the dynamic interdependence of linked aquatic and terrestrial systems (Piovia‐Scott et al. 2016; George and Collins 2023). Future research to more clearly identify the connections between lakeshore riparian condition and nearshore littoral habitat structural characteristics, functional diversity, and ecological relationships is key to identifying effective strategies for protecting adfluvial coastal cutthroat trout in small lakes on the forest land base.

## Author Contributions

**Tracy Michalski:** conceptualization (equal), data curation (equal), formal analysis (equal), funding acquisition (lead), investigation (equal), methodology (equal), project administration (lead), resources (lead), software (equal), supervision (equal), validation (equal), visualization (equal), writing – original draft (equal), writing – review and editing (equal). **Heather Klassen:** conceptualization (equal), data curation (equal), formal analysis (equal), investigation (equal), methodology (equal), project administration (supporting), software (equal), validation (equal), visualization (equal), writing – original draft (equal), writing – review and editing (equal). **Peter Ott:** conceptualization (equal), data curation (equal), formal analysis (equal), methodology (equal), software (equal), validation (equal), writing – review and editing (supporting). **Carl Schwarz:** conceptualization (supporting), data curation (equal), formal analysis (equal), methodology (equal), software (equal), validation (equal), writing – review and editing (supporting).

## Conflicts of Interest

The authors declare no conflicts of interest.

## Data Availability

Data used in the analyses for this study are available at Science Data Bank https://www.scidb.cn.

## Appendix A

**TABLE A1:** Average monthly surface temperature (°C) above the thermocline and dissolved oxygen (mg/L) at Cedar, Jessie, Lost, Mud, Nola, and Spirit Lakes on Central Vancouver Island, British Columbia, Canada June–September 2017.

Lake	Month											
	June			July			August			September		
	Temp. (°C)	DO (mg/L)	TDS (ppm)	Temp. (°C)	DO (mg/L)	TDS (ppm)	Temp. (°C)	DO (mg/L)	TDS (ppm)	Temp. (°C)	DO (mg/L)	TDS (ppm)
Nola	14.7	9.5	11.8	17.5	9.1	24.9	19.2	8.8	61.5	14.8	9.5	43.0
Spirit	17.7	9.2	9.73	22.1	8.6	22.8	21.9	8.6	54.3	18.9	8.7	36.6
Lost	17.4	8.9	18.6	20.0	—	—	23.0	8.1	42.8	16.9	8.9	66.1
Jessie	14.2	10.1	17.0	19.1	9.0	39.1	21.0	8.8	36.3	16.4	8.9	63.1
Cedar	18.3	—	11.1	22.1	8.1	24.3	22.1	8.2	59.4	19.4	8.1	38.5
Mud	17.8	—	11.1	21.5	8.7	24.6	21.3	8.6	59.5	19.9	8.8	39.3

**TABLE A2:** Distribution (percent) of forest age classes (years) within 20 m of shore, percent riparian (within 20 m of shore), watershed area and percent watershed harvested (ha) and years of harvest at Nola, Spirit, Lost, Jessie, Cedar, and Mud Lakes on Central Vancouver Island, British Columbia, Canada. Forest age and harvest year data are from the Government of British Columbia Data Catalog (https://catalogue.data.gov.bc.ca/). Percentages of each age class were estimated within a 20 m boundary around the lake perimeter. Lakes are presented from lowest to highest percent of forest harvest within 20 m of shore.

Lake	Distribution (percent) of forest age classes (years) within 20 m of shore									Percent of forest harvest within 20 m of shore	Watershed area (ha)	Percent of watershed harvested (ha)	Years harvested
	1–20	21–40	41–60	61–80	81–100	101–120	121–140	141–250	250+				
Nola	—	—	—	—	—	—	—	—	100	0	568	0	N/A
Spirit	—	—	—	—	—	—	—	—	100	0	379	31.7	2014–2017
Lost	50	—	—	—	—	—	—	—	50	50	87	16.8	2010
Jessie	—	35	—	40	—	—	—	—	25	75	321	32.8	1975–1986
Cedar	—	5	—	46	22	7	—	—	20	80	335	37.8	1947–2015
Mud	2	—	—	22	42	8	—	26	—	100	246	41.4	1948–2013

**TABLE A3:** Vegetation and decaying wood average percent cover of each vertical layer by vegetation category in aquatic and terrestrial plots at Nola, Spirit, Lost, Jessie, Cedar, and Mud Lakes. Vertical layers of the aquatic plots are categorized as overhanging (growing above lake surface), floating (floating on the lake surface), or submerged (below lake surface), and vertical layers of terrestrial plots were characterized by height (> 10 m [overstory], 2–10 m [shrub], < 2 m [understory]). Decaying wood is ≥ 10 cm diameter.

Vertical layer and vegetation category	Average percent cover					
	Nola	Spirit	Lost	Jessie	Cedar	Mud
Aquatic plots						
Overhanging						
Evergreen tree	35	7	1	7	0	0
Deciduous tree	0	0	0	9	0	0
Evergreen shrub	0	0	0	0	0	0
Deciduous shrub	37	5	1	5	2	2
Herb	3	< 1	20	21	0	12
Total	75	12	22	42	2	14
Floating						
Evergreen tree	0	0	0	0	0	0
Deciduous tree	0	0	0	0	0	0
Evergreen shrub	0	0	0	0	0	0
Deciduous shrub	0	0	0	0	< 1	0
Herb	< 1	19	39	< 1	15	9
Total	< 1	19	39	< 1	16	9
Submerged						
Evergreen tree	0	< 1	0	0	0	0
Deciduous tree	0	0	0	0	0	0
Evergreen shrub	0	1	0	0	0	0
Deciduous shrub	0	< 1	0	0	< 1	0
Herb	31	2	22	< 1	13	< 1
Total	31	3	22	< 1	13	< 1
Decaying wood	18	10	10	6	11	9
Terrestrial plots						
Overstory (> 10 m)						
Evergreen tree	87	57	35	55	41	19
Deciduous tree	0	0	29	0	0	13
Evergreen shrub	0	0	0	0	0	0
Deciduous shrub	0	0	0	0	0	0
Herb	0	0	0	0	0	0
Total	87	57	64	55	41	32
Shrub (2–10 m)						
Evergreen tree	21	8	11	14	19	16
Deciduous tree	0	0	0	7	0	14
Evergreen shrub	0	0	0	0	0	1
Deciduous shrub	15	16	7	9	0	3
Herb	0	0	0	0	0	1
Total	36	24	18	30	19	35
Understory (< 2 m)						
Evergreen tree	5	1	1	7	2	2
Deciduous tree	0	0	< 1	0	0	1
Evergreen shrub	6	24	16	5	24	18
Deciduous shrub	13	34	11	33	18	29
Herb	9	20	50	23	27	32
Total	33	79	78	68	71	82
Decaying wood	8	13	0	0	2	0

**TABLE A4:** Estimated Pearson's correlation coefficients (*r*), *t*‐statistics (*t*), and *p*‐values (*p*) for the relationship between aquatic and terrestrial vegetative layers (weighted average percent cover) at Nola, Spirit, Lost, Jessie, Cedar, and Mud Lakes on Vancouver Island, British Columbia, Canada. The degrees of freedom (df) equals 4 in all cases. Note the smaller the *p*‐value, the more significant the relationship (Obilor and Amadi 2018).

Aquatic	Terrestrial								
	Overstory			Shrub			Understory		
	rcorr	tstat	pvalue	rcorr	tstat	pvalue	rcorr	tstat	pvalue
Overhanging	0.81	2.798	0.0489	0.63	1.635	0.1774	−0.88	−3.66	0.0216
Floating	−0.06	−0.119	0.9113	−0.81	−2774	0.0501	0.54	1.277	0.2706
Submerged	0.76	2.348	0.0786	−0.08	−0.167	0.8756	−0.69	−1.914	0.1281

**TABLE A5:** Number and minimum, maximum and mean (in parentheses) fork lengths (mm) of cutthroat captured June–September 2017 at Nola, Spirit, Lost, Jessie, Cedar, and Mud Lakes on Vancouver Island, British Columbia, Canada.

Age	Nola		Spirit		Lost		Jessie		Cedar		Mud	
	*n*	Length	*n*	Length	*n*	Length	*n*	Length	*n*	Length	*n*	Length
1 YO	27	130–185 (151.9)	2	210–230 (220.0)	6	127–175 (156)	0	—	0	—	3	95–127 (107.0)
2 YO	11	180–260 (210.2)	6	220–255 (241.3)	27	150–253 (205.2)	4	171–230 (199.0)	3	200–250 (233.3)	14	189–255 (277.2)
3 YO	10	235–295 (257.6)	6	236–275 (252.2)	18	210–280 (245)	7	220–290 (258.7)	3	250–280 (268.3)	10	229–300 (255.0)
4 YO	3	300–335 (311.7)	0	—	2	249–254 (251.5)	4	275–345 (300.0)	6	280–310 (297.0)	4	275–305 (291.7)
5 YO	1	296	0	—	0	—	2	310–390 (350.0)	2	305–340 (322.5)	4	310–325 (318.7)
6 YO	2	320–350 (335.0)	0	—	0	—	1	350	0	—	0	—

**TABLE A6:** Proportions of the most common and most dominant (by wet weight) prey items in diets of cutthroat sampled from Nola Lake on Central Vancouver Island, British Columbia, Canada. Values for most common taxa represent the proportion of fish diets in which each prey taxon appears. Values for most dominant taxa represent the proportion of diets in which the prey item contributed the greatest biomass.

Lake and year class	Most common	Most dominant
Nola		
Juveniles		
1YO (37 fish)	Hymenoptera (0.35)	Hymenoptera (0.72)
	Aquatic‐Other (0.32)	Diptera‐Terrestrial (0.20)
	Terrestrial‐Other (0.18)	Aquatic‐Other (0.05)
	Diptera‐Terrestrial (0.08)	Terrestrial‐Other (0.02)
	Chironomidae (0.05)	Chironomidae (0.01)
2YO (20 fish)	Aquatic‐Other (0.4)	Hymenoptera (0.93)
	Hymenoptera (0.25)	Terrestrial‐Other (0.04)
	Terrestrial‐Other (0.25)	Aquatic‐Other (0.02)
	Diptera‐Terrestrial (0.1)	Diptera‐Terrestrial (< 0.01)
3YO (15 fish)	Aquatic‐Other (0.53)	Hymenoptera (0.89)
	Terrestrial‐Other (0.33)	Terrestrial‐Other (0.07)
	Hymenoptera (0.13)	Aquatic‐Other (0.03)
Adults		
4YO (2 fish)	Terrestrial‐Other (0.5)	Terrestrial‐Other (0.96)
	Aquatic‐Other (0.5)	Aquatic‐Other (0.03)
5YO (1 fish)	Aquatic‐Other (1)	Aquatic‐Other (1)
Spirit		
Juveniles		
2YO (2 fish)	Aquatic‐Other (0.5)	Aquatic‐Other (0.63)
	Ephemeroptera (0.5)	Ephemeroptera (0.36)
3YO (8 fish)	Aquatic‐Other (0.37)	Aquatic‐Other (0.48)
	Terrestrial‐Other (0.37)	Cladocera (0.42)
	Cladocera (0.12)	Terrestrial‐Other (0.07)
	Ephemeroptera (0.12)	Ephemeroptera (0.01)
Lost		
Juveniles		
2YO (22 fish)	Aquatic‐Other (0.42)	Aquatic‐Other (0.37)
	Terrestrial‐Other (0.19)	Chironomidae (0.35)
	Chironomidae (0.14)	Terrestrial‐Other (0.20)
	Fish (0.14)	Hymenoptera (0.02)
	Diptera‐Terrestrial (0.04)	Fish (0.02)
	Hymenoptera (0.04)	Diptera‐Terrestrial (< 0.01)
3YO (14 fish)	Aquatic‐Other (0.57)	Cladocera (0.77)
	Diptera‐Terrestrial (0.21)	Aquatic‐Other (0.19)
	Chironomidae (0.07)	Diptera‐Terrestrial (0.01)
	Cladocera (0.07)	Chironomidae (< 0.01)
	Fish (0.07)	Fish (< 0.01)
Adults		
4YO (2 fish)	Aquatic‐Other (0.5)	Aquatic‐Other (0.52)
	Terrestrial‐Other (0.5)	Terrestrial‐Other (0.47)
Jessie		
Juveniles		
2YO (6 fish)	Aquatic‐Other (0.66)	Acari (0.50)
	Acari (0.16)	Aquatic‐Other (0.35)
	Terrestrial‐Other (0.16)	Terrestrial‐Other (0.13)
3YO (15 fish)	Aquatic‐Other (0.40)	Diptera‐Terrestrial (0.43)
	Terrestrial‐Other (0.26)	Aquatic‐Other (0.25)
	Chironomidae (0.06)	Ephemeroptera (0.11)
	Diptera‐Aquatic (0.06)	Terrestrial‐Other (0.10)
	Diptera‐Terrestrial (0.06)	Chironomidae (0.04)
	Ephemeroptera (0.06)	Trichoptera (0.04)
	Trichoptera (0.06)	Diptera‐Aquatic (< 0.01)
Adults		
4YO (7 fish)	Aquatic‐Other (0.42)	Gastropoda (0.64)
	Chironomidae (0.14)	Aquatic‐Other (0.27)
	Diptera‐Terrestrial (0.14)	Terrestrial‐Other (0.06)
	Gastropoda (0.14)	Chironomidae (0.01)
	Terrestrial‐Other (0.14)	Diptera‐Terrestrial (< 0.01)
5YO (4 fish)	Aquatic‐Other (0.50)	Gastropoda (0.88)
	Gastropoda (0.25)	Aquatic‐Other (0.10)
	Terrestrial‐Other (0.25)	Terrestrial‐Other (< 0.01)
6YO (2 fish)	Fish (0.50)	Terrestrial‐Other (0.66)
	Terrestrial‐Other (0.50)	Fish (0.33)
Cedar		
Juveniles		
2YO (4 fish)	Copepoda (0.50)	Copepoda (0.50)
	Cladocera (0.50)	Cladocera (0.49)
3YO (4 fish)	Cladocera (0.50)	Cladocera (0.99)
	Aquatic‐Other (0.25)	Aquatic‐Other (< 0.01)
	Chironomidae (0.25)	Chironomidae (< 0.01)
Adults		
4YO (6 fish)	Cladocera (0.33)	Cladocera (0.96)
	Chironomidae (0.16)	Copepoda (0.02)
	Copepoda (0.16)	Chironomidae (< 0.01)
	Diptera‐Aquatic (0.16)	Diptera‐Aquatic (< 0.01)
	Fish (0.16)	Fish (< 0.01)
5YO (2 fish)	Cladocera (0.50)	Cladocera (0.84)
	Aquatic‐Other (0.50)	Aquatic‐Other (0.15)
Mud		
Juveniles		
2YO (4 fish)	Cladocera (0.50)	Cladocera (0.99)
	Aquatic‐Other (0.25)	Aquatic‐Other (< 0.01)
	Chironomidae (0.25)	Chironomidae (< 0.01)
3YO (8 fish)	Cladocera (0.50)	Cladocera (0.97)
	Aquatic‐Other (0.25)	Aquatic‐Other (0.02)
	Diptera‐Terrestrial (0.12)	Diptera‐Terrestrial (< 0.01)
	Fish (0.12)	Fish (< 0.01)
Adults		
4YO (1 fish)	Cladocera (1)	Cladocera (1)
5YO (4 fish)	Terrestrial‐Other (0.50)	Cladocera (0.95)
	Chironomidae (0.25)	Terrestrial‐Other (0.02)
	Cladocera (0.25)	Chironomidae (0.01)

**TABLE A7:** Estimated Pearson's correlation coefficients (rcorr), *t*‐statistics (tstat), degrees of freedom (df), and *p*‐values (pvalue) for the relationship between intake of aquatic and terrestrial invertebrates (wet mass) and the percent vegetation in the aquatic and terrestrial strata by juvenile and adult cutthroat trout captured June–September 2017 at Nola, Spirit, Lost, Jessie, Cedar, and Mud Lakes on Vancouver Island, BC. Note the smaller the *p*‐value, the more significant the relationship (Obilor and Amadi 2018).

Strata	Juveniles (1YO, 2YO, 3YO)							
	Aquatic wet mass				Terrestrial wet mass			
	*r*corr	*t*stat	df	*p*value	*r*corr	*t*stat	df	*p*value
Aquatic								
Overhanging	−0.44	−0.973	4	0.3859	0.89	3.82	4	0.0188
Floating	0.07	0.15	4	0.8882	−0.45	−1.004	4	0.3721
Submerged	0.11	0.224	4	0.8334	0.73	2.132	4	0.0999
Decaying wood	0.07	0.143	4	0.8935	0.87	3.614	4	0.0225
Terrestrial								
Overstory	−0.37	−0.793	4	0.4722	0.81	2.813	4	0.0482
Shrub	−0.48	−1.08	4	0.341	0.55	1.304	4	0.2623
Understory	0.04	0.082	4	0.9384	−0.96	−6.995	4	0.0022
Decaying wood	−0.31	−0.654	4	0.549	0.35	0.738	4	0.5016

Strata	Adults (4YO, 5YO, 6YO)							
	Aquatic wet mass				Terrestrial wet mass			
	*r*corr	*t*stat	df	*p*value	*r*corr	*t*stat	df	*p*value
Aquatic								
Overhanging	0.17	0.299	3	0.7847	0.72	1.796	3	0.1704
Floating	−0.42	−0.793	3	0.4859	−0.63	−1.398	3	0.2565
Submerged	−0.54	−1.12	3	0.3442	−0.09	−0.162	3	0.8819
Decaying wood	−0.59	−1.27	3	0.2936	−0.06	−0.102	3	0.9255
Terrestrial								
Overstory	−0.05	−0.087	3	0.936	0.47	0.931	3	0.4204
Shrub	0.1	0.177	3	0.8705	0.45	0.868	3	0.4491
Understory	0.06	0.11	3	0.9193	−0.49	−0.982	3	0.3985
Decaying wood	−0.33	−0.605	3	0.5881	0.24	0.435	3	0.6928
